# Neuroinflammatory-related biomarkers and psychiatric symptom changes in substance use disorder: a South African cohort study

**DOI:** 10.1186/s12888-026-08237-0

**Published:** 2026-05-30

**Authors:** Alexio Dos Santos, Christiaan B. Brink, Esmé Jansen van Vuren

**Affiliations:** 1 Centre of Excellence of Pharmaceutical Sciences, North-West University, Potchefstroom, South Africa; 2 Hypertension in Africa Research Team (HART), North-West University, Potchefstroom, 2520 South Africa; 3 South African Medical Research Council Unit for Hypertension and Cardiovascular Disease, North-West University, Potchefstroom, South Africa

**Keywords:** Substance use disorder, Biomarkers, Psychiatric symptoms, Kynurenine pathway, South Africa

## Abstract

**Background:**

Substance use disorder (SUD) is a debilitating neuropsychiatric condition characterized by persistent compulsive drug use and associated cognitive and psychiatric impairments. Emerging evidence suggests that neuroinflammatory pathways contribute to the pathophysiology of SUD. However, the relationships between neuroinflammatory-related biomarkers and psychiatric symptomatology in SUD remain poorly understood. This study aimed to investigate neuroinflammatory-related biomarkers and their association with psychiatric symptom changes in individuals with SUD.

**Methods:**

Participants with SUD (*N* = 100) at three local facilities in the North West Province of South Africa were enrolled. At baseline and following three weeks of treatment, psychiatric symptoms were assessed using the Brief Psychiatric Rating Scale (BPRS). Serum levels of superoxide dismutase (SOD), glutathione (GSH), glutathione disulphide (GSSG), tryptophan, kynurenine (KYN), kynurenic acid (KYNA), serotonin (5-HT), 5-hydroxyindoleacetic acid (5-HIAA), cortisol and serum S100 calcium-binding protein B (S100B) were measured.

**Results:**

Over the 3-week treatment period BPRS scores and serum levels of tryptophan, 5-HT, 5-HIAA, KYN, KYNA, cortisol and GSH decreased, whereas SOD, S100B and GSSG levels increased. Baseline BPRS correlated with baseline serum levels of 5-HIAA, KYNA and GSSG. A decrease in BPRS correlated with baseline levels of 5HIAA, KYN and KYNA, as well as with 3-week changes in levels of select biomarkers (∆tryptophan, ∆5-HT, ∆5-HIAA, ∆KYN, ∆KYNA, ∆GSSG and ∆cortisol).

**Conclusion:**

The results suggest that baseline 5-HIAA, KYN and KYNA may predict psychiatric symptom improvement whereas tryptophan, 5-HT, 5-HIAA, KYN, KYNA, GSSG and cortisol may explain the neurobiological basis of changed psychiatric symptomatology.

**Supplementary Information:**

The online version contains supplementary material available at 10.1186/s12888-026-08237-0.

## Introduction

The abuse of substances is a global issue and a persistent health problem worldwide [1]. The United Nations Office on Drugs and Crime (UNODC) reported that 275 million people aged 15–64 have used substances at least once [2], with the incidence of substance use disorder (SUD) rising dramatically between 2010 and 2019 [3]. Additionally, the World Health Organization (WHO) estimated that over 300 000 deaths in 2016 were due to alcohol and SUDs [4, 5]. South Africa is one of the world’s largest producers of several substances associated with SUD, such as cannabis [6, 7]. According to the South African Community Epidemiology Network on Drug Use (SACENDU) report, alcohol, cannabis, cocaine, and heroin/opiates were the most common substances of abuse in the North West province in South Africa from 2018 to June 2020 [6], with polysubstance abuse representing 69% of all the SUD cases in the North West province [7].

Substance use disorder (SUD) involves a physical, mental, and emotional compulsion to use mind-altering substances like drugs or alcohol [8], often leading to increased tolerance and higher doses to achieve the desired effect. Due to its complexity and associated medical conditions, SUD treatment is typically managed separately from general care [9]. The Diagnostic and Statistical Manual of Mental Disorders (DSM-5^®^) describe essential features of SUD during use and withdrawal as a collection of unwanted cognitive, behavioural, and physiological symptoms, ultimately causing the individual to continue using the substances despite negative consequences [10]. When substance use is abruptly stopped, unpleasant or even dangerous withdrawal symptoms follow, spurring further use to avoid withdrawal effects, eventually leading to a persisting cycle of continued use [10, 11].

Substance use disorder (SUD) is a condition that progresses through a deteriorating three-phase cycle: binge and intoxication, withdrawal and negative effects, and preoccupation and anticipation (craving) [12]. Ultimately, these three phases are mediated by complex neural circuits involving both the limbic system and the prefrontal cortex [12, 13]. Three key limbic regions including the amygdala, hippocampus, and nucleus accumbens are primarily involved in regulating reward, emotion, and punishment [14]. Furthermore, the mesocorticolimbic dopaminergic pathways, which project from the ventral tegmental area (VTA) to the nucleus accumbens and to the medial and dorsal prefrontal cortex, respectively, play a central role in the behavioural manifestations of addiction, including drug craving, dependence, and relapse [13].

Due to the complex and heterogeneous neurobiological basis of SUD, it is often difficult to determine why some individuals respond to treatment while others do not. Peripheral biomarkers may provide valuable insights into the neurobiological underpinnings of SUD and the associated dynamic changes in symptomatology, ultimately accompanying disease progression and treatment response. An increasing body of evidence suggest that modulation of inflammatory mediators plays a role in the onset and persistence of SUD and may serve as promising diagnostic and therapeutic targets [15]. In this regard, chronic neuroinflammation has been associated with neuroplastic and neurochemical alterations within dopaminergic and limbic pathways that underlie craving and relapse. These inflammatory changes often occur in parallel with disruptions in stress-related systems (e.g., cortisol), oxidative stress (superoxide dismutase (SOD), reduced glutathione (GSH), and glutathione disulphide (GSSG)), tryptophan metabolism through the kynurenine pathway, and astroglial activation markers such as serum calcium-binding protein B (S100B). Measuring these biomarkers may improve our understanding of the neurobiological basis, diagnosis, and ultimately the treatment of SUD [16–21].

To our knowledge, no clinical study has assessed these biomarkers in relation to psychiatric symptomatology in a South African population with SUD. Therefore, this study aims to establish whether baseline biomarker levels predict improvement in psychiatric symptomatology (assessed with the Brief Psychiatric Rating Scale (BPRS)) after a three-week in-patient treatment period in a cohort of South African individuals with SUD.

## Methods

### Study layout

Based on a power analysis (estimated effect size of F = 0.42 and a minimum clinical difference of 15%, a minimum sample size of 61 was calculated to be sufficient to obtain a 90% power on a 5% level of significance), this study consisted out of a SUD cohort of 102 participants at baseline who have been admitted (between 2020 and 2021) to any of three identified state (public) and private rehabilitation facilities in the Kenneth Kaunda district in the North West province of South Africa. At baseline, 53 participants were recruited from the public sector facility (Site 1), 47 from a non-governmental organisation in the private sector (Site 2), and 2 from a second private-sector facility (Site 3). Following a 3-week treatment period, 79 participants were successfully followed up, including 42 from Site 1, 36 from Site 2, and 1 from Site 3. This study assessed changes in biomarker profiles and psychiatric symptoms in eligible individuals occurring between admission and a facility-specific standardized 3-week treatment phase. While minor site-specific variations were observed, the overall treatment structure consistently followed a staged detoxification approach across facilities. Briefly, in Week 1, patients underwent a comprehensive assessment, including vital signs, blood glucose, injury screening, and routine investigations (blood and urine tests), with diagnosis based on DSM-5 criteria. Initial treatment, such as intravenous saline, diazepam, and vitamin supplementation (B12, C, multivitamins, magnesium), was tailored to individual clinical needs, as responses to detoxification varied. In Week 2, patients received continued monitoring of vital signs and glucose levels. By Week 3, discharge preparation included repeat blood tests and supplementation with thiamine, vitamin B-complex, and B12. Each participant served as their own control, with relevant measurements taken at baseline (upon admission) and again after three weeks, marking the end of the standardised treatment period and the conclusion of the clinical phase of the study.

### Inclusion, exclusion criteria and recruitment

#### Inclusion criteria

Participants were required to have a diagnosis of SUD, be between 18 and 65 years of age, of any ethnicity, and included both men and women. Participants also needed to be competent and willing to provide informed consent. Participants with comorbid psychiatric disorders were not excluded, as such symptoms often overlap with those induced by substance use. Moreover, psychiatric symptom changes associated with substance use were the primary outcome of interest in this study.

#### Exclusion criteria

Participants were excluded if they tested positive for COVID-19 upon admission (as the study took place during the pandemic), had a diagnosed neurological disorder, head trauma, or intellectual disability (i.e., without capacity to consent), were taking corticosteroids or drugs affecting steroid metabolism, were pregnant or breastfeeding, displayed aggressive behaviour that impeded recruitment, or had a tympanic temperature above 37.5 °C (as fever or immune responses could influence biomarker levels).

Recruitment procedures were done in similar manner by the three facilities in accordance with the standardized operating procedures of the facilities. Patients eligible for participation were introduced to the procedures of the study (including information regarding the study objectives, outcomes, potential benefits and risks, procedures and expectations, as well as voluntary participation and freedom to withdraw) by either a registered nurse or psychiatrist at the respective facilities, in English or the patient’s home language. If the patient gave verbal consent to participate in the study, written informed consent was obtained before any data and samples were collected. This process was repeated for all participants at the 3-week follow-up phase. If a patient chose to withdraw at any time during the study, he/she was allowed to freely do so without any impact on standard treatment. Patient confidentiality was maintained through private consultations and the pseudonymisation of data. Each participant was assigned a unique identification number (coded deidentification), which only the treating psychiatrist could link to the patient’s identity.

### Sociodemographic information and medical history

Sociodemographic information was collected from the patient files at the respective facilities and included age, sex, ethnicity, employment status, level of education, and household income. Socioeconomic status (SES) was calculated using a point system that was adapted from Kuppuswamy’s Socioeconomic Status Scale for a South African environment, scoring participants in 3 categories: employment, education, and household income. Scores were put into categories according to low (5–18), middle (19–24) and high (25–30) socioeconomic groups. Medical history included a family history of SUD and details on the types of substances used by the patient. This information was collected by the respective facilities through patient self-report at the time of admission. Commonly reported substances included alcohol, cannabis, opioids and benzodiazepines with majority of participants reporting concurrent use of multiple substances (i.e., being polysubstance users).

### Biomarker analysis

Human superoxide dismutase (SOD) was analysed with the enzyme-linked immunosorbent assay (ELISA) kit according to the manufacture’s manual (Elabscience, distributed by Biocom Africa, Johannesburg, South Africa) and human S100B was analysed with the ELISA kit according to the manufacture’s manual (Merck, South Africa). Cortisol, GSH, GSSG and the kynurenine pathway’s monoamines (tryptophan, 5-HT, 5-HIAA, KYN, KYNA, quinolinic acid) were measured by a liquid chromatography mass spectrometry (LCMS) method as described before [22].

### Psychiatric assessment

Overall psychiatric distress and five factors of psychiatric symptoms [positive (e.g., hallucinations), negative (e.g., emotional withdrawal), depression (e.g., depressed mood), mania (e.g., elevated mood), and disorientation (e.g., disorganized speech)] were assessed using the BPRS. BPRS scores for the patient’s psychiatric symptoms were assigned by the treating psychiatrist, ranging from 0 (not present) to 7 (very severe) [23].

### Statistical analysis

**Fig. 1:**
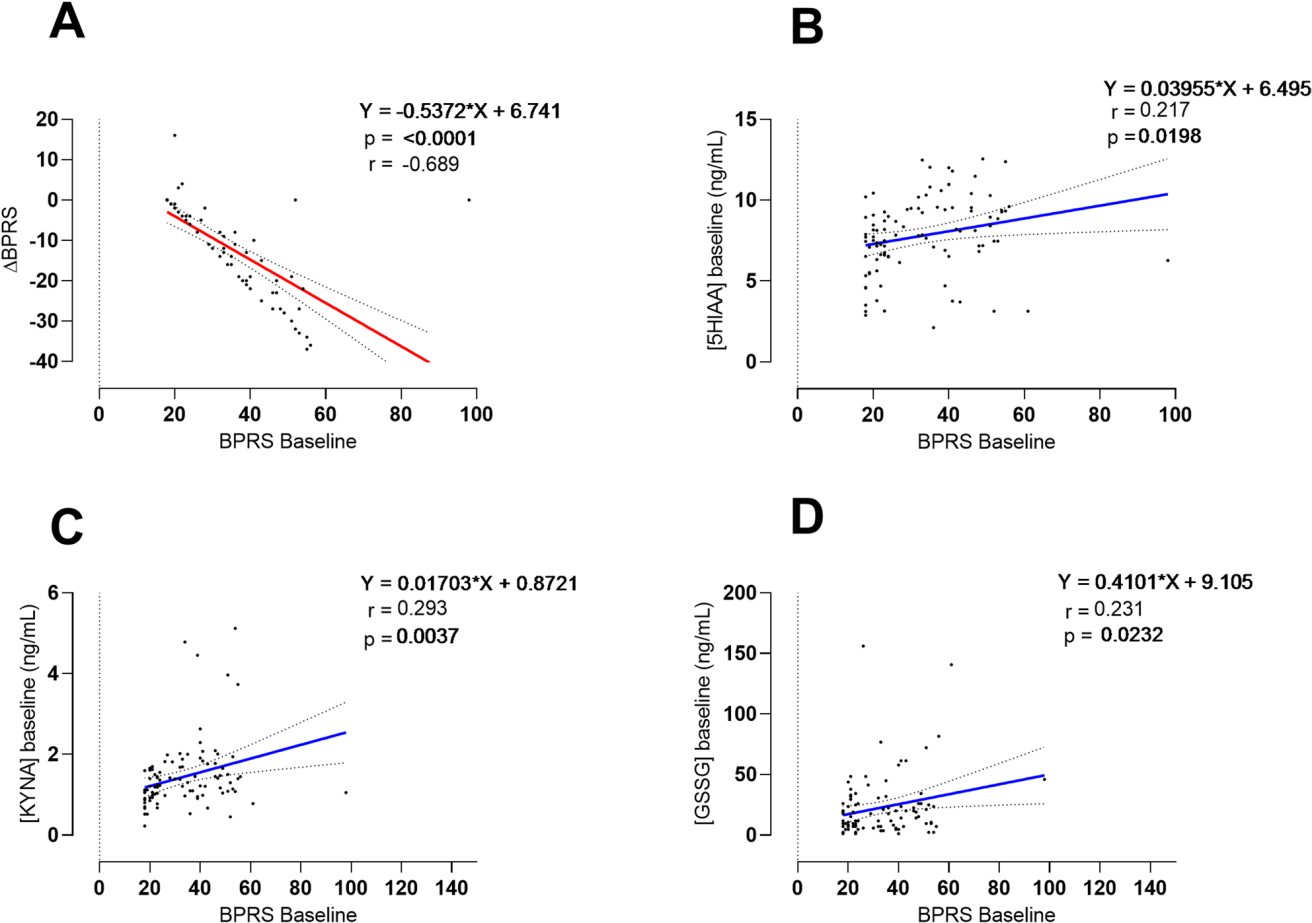
A correlation between BPRS scores at baseline and biomarker levels at baseline. Simple linear regression to determine the correlation between BPRS scores at baseline with **A.** ∆BPRS score, **B** 5-HIAA baseline serum concentration, **C** KYNA baseline serum concentration, and **D** GSSG baseline serum concentration. In this figure ∆ refers to change over 3-weeks (data at follow-up minus data at baseline), *BPRS = Brief Psychiatric Rating Scale*,* KYNA = kynurenic acid; GSSG = glutathione disulphide; 5-HIAA = 5-hydroxyindoleacetic acid*

**Fig. 2:**
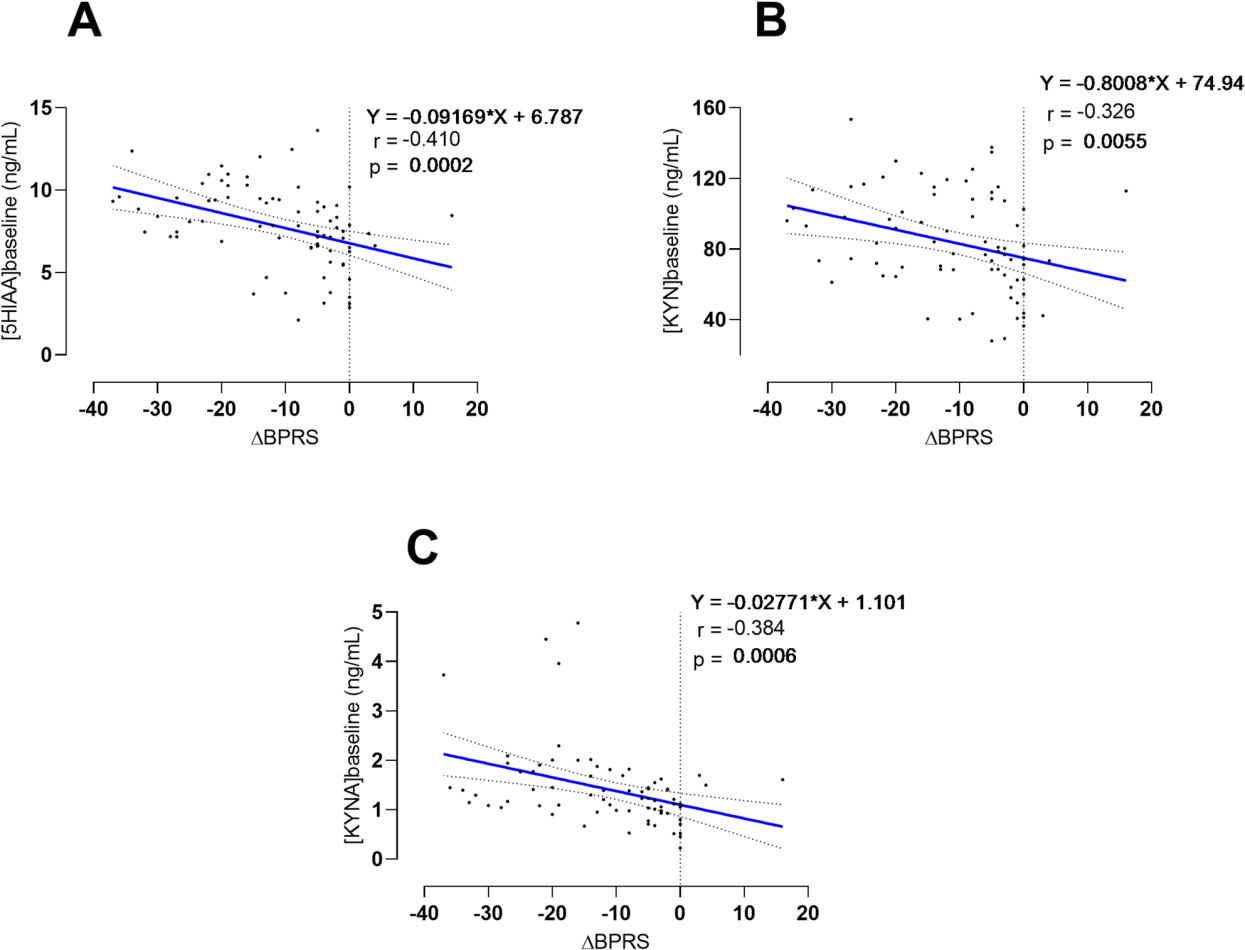
A comparison of ∆BPRS scores and biomarker levels at baseline. Simple linear regression comparing ∆BPRS with **A** 5-HIAA baseline serum concentration, **B** KYN baseline serum concentration, **C** KYNA baseline serum concentration. In this figure ∆ refers to change over 3-weeks (data at follow-up minus data at baseline). *BPRS = Brief Psychiatric Rating Scale*,* KYN = kynurenine; KYNA = kynurenic acid; 5-HIAA = 5-hydroxyindoleacetic acid*

**Fig. 3:**
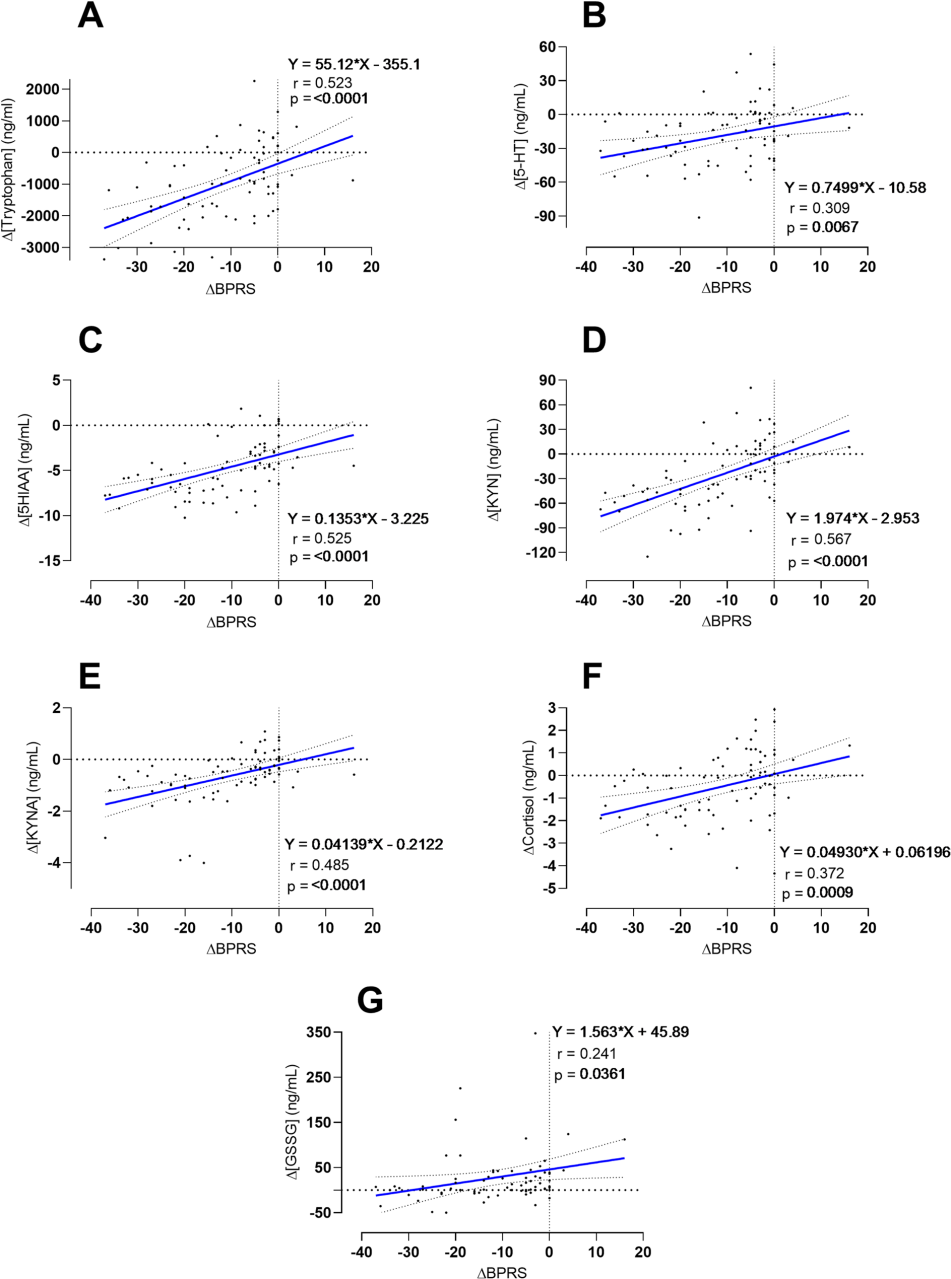
A comparison of ∆BPRS scores and ∆ biomarker levels. Simple linear regression comparing ∆BPRS with **A** ∆Tryptophan serum concentration over time, **B** ∆5-HT serum concentration over time, **C** ∆5-HIAA serum concentration over time, **D** ∆KYN serum concentration over time, **E** ∆KYNA serum concentration over time, **F** ∆cortisol serum concentration over time, **G** ∆GSSG serum concentration over time, *where ∆ refers to change over 3-weeks (data at follow-up minus data at baseline); BPRS = Brief Psychiatric Rating Scale; KYN = kynurenine*,* KYNA = kynurenic acid; GSSG = glutathione disulphide; 5-HIAA = 5-hydroxyindoleacetic acid*,* 5-HT = serotonin*

All statistical analyses were performed with GraphPad Prism^®^ version 9.2.0 and Statistical Package for the Social Sciences (SPSS^®^) version 27. Variables were tested for normality by making use of quantile-quantile (Q-Q) plots. Descriptive statistics and paired t-tests were performed for baseline and follow-up samples. Categorical data are reported as frequencies and percentages. The mean ± 95% confidence interval (CI) are reported for normally distributed variables, and the median (25th and 75th quartile) are reported for variables that follow a skewed distribution. Data of the ∆ mean represent the difference between values at baseline versus follow-up (as seen in Tables 1 and 2; Figs. 1 and 2, and Fig. 3). The paired t-test results are indicated with the Cohen’s d (d) value indicates effect size (practical significance) of the associated change over three weeks (∆ mean), where 0.2 to 0.49 indicates a small effect size, 0.5 to 0,79 indicates a medium effect size and ≥ 0.8 indicates a large effect size. Also, note that with the Cohen’s d value an asterisk indicates a statically significant difference between the values of baseline and at follow-up, where * indicates *p* < 0.05, ** indicates *p* < 0.01 and *** indicates *p* < 0.001. Between-site comparisons of Site 1 and Site 2 were performed using independent-samples t-tests. Pearson correlations were used to identify correlations between biomarkers at baseline and ∆biomarkers over time together with the baseline BPRS and ∆BPRS, and partial correlations while adjusting for confounders. For interpretation of the statistical analyses *p* < 0.05 was regarded significant; *r* < 0.2 represents very weak correlation; *r* = 0.2 to 0.39 represents weak correlation, *r* = 0.4 to 0.59 represents moderate correlation; *r* > 0.6 to 0.79 represents strong correlation (as seen in Tables 1 and 2; Figs. 1, 2 and 3).

### Ethical considerations

This study was approved by the North-West University Health Research Ethics Committee (NWU-HREC) of the Faculty of Health Sciences (ethics approval no. NWU-00449-20-A1). Approval was also obtained by the North West Department of Health Research Committee (NWDoHRC) as well as by the clinical manager of the respective rehabilitation facilities. The study approval and execution have been conducted according to the applicable ethical guidelines and principles of relevant South African national legislation, as governed by the Ethics in Health Research: Principles, Processes and Structures guidelines, 2nd ed., 2015, of the National Department of Health in South Africa (version at the time the study was conducted).

## Results

The general demographics of the study population are presented in Table 1. This study population were mostly represented by men (90.6%) of Black African ethnicity (82.3%), mostly young adults with a median age of 28, ranging from 18 to 65 (interquartile range = 13). Patients from the three different facilities received different treatment regimens, as per its own standard treatment guideline, ranging from paracetamol and thiamine as a detoxification strategy, to antipsychotic medication such as haloperidol, clozapine, and phenytoin.

**Table 1:** General demographics of the study population at baseline, including the number of participants (*n* values) and percentage of the study population)

Variable	At Baseline
Male	*n* = 87 (90.6%)
Black African	*n* = 79 (82.3%)
White	*n* = 15 (15.6%)
Low Socio-economic score (SES)*	*n* = 90 (94.7%)
Middle Socio-economic score *	*n* = 5 (5.3%)
High Socio-economic score	*n* = 0 (0%)
Mother with SUD	*n* = 6 (6.3%)
Father with SUD	*n* = 8 (8.4%)
Chronic Medication use	*n* = 43 (45.3%)
Age (years)	Median: 28; IQR: 13
Body Mass (kg)	Mean: 59.21 ± 1.74

SUD = substance use disorder

*SES was calculated using a point system that was adapted from Kuppuswamy’s Socioeconomic Status Scale for a South African environment, scoring participants in 3 categories: employment, education, and household income. Scores were put into categories according to low (5–18), middle (19–24) and high (25–30) socioeconomic groups [24]

The psychometric scores from the BPRS and biomarker serum concentrations of the study population at baseline, 3-week follow-up ∆ mean are presented in Table 2. Significant decreases between baseline and 3-week follow-up were observed for the BPRS score (*p* < 0.001), tryptophan (*p* < 0.001), 5-HT (*p* < 0.001), 5-HIAA (*p* < 0.001), KYN (*p* < 0.001), KYNA (*p* < 0.001), GSH (*p* = 0.013) and cortisol (*p* = 0.003). In contrast, significant increases were shown for SOD (*p* = 0.023), S100B (*p* = 0.040) and GSSG (*p* < 0.001) from baseline to follow-up. Removal of outliers from these paired *t*-test analyses, as identified by the ROUT method, did not change the outcome of the study results, and we hence did not exclude any data points in the final analyses as presented in Table 2.

Of the values with statistically significant differences indicated above, a large effect size was evident only for the change in BPRS score (d = -0.989) and the change in 5-HIAA serum level (d = -1.620) over the 3-week treatment period. Medium effect sizes were seen for the change in serum levels of tryptophan (d = -0.812), 5-HT (d = 0.693), KYN (d = -0.581) and KYNA (d = -0.718), whereas only a small effect size was seen for the change in serum levels of SOD (d = 0.260), S100B (d = 0.232), cortisol (d = -0.343), GSH (d = -0.283) and GSSG (d = 0.412).

**Table 2:** BPRS scores and measured blood serum concentrations of biomarkers of the study population at baseline and follow-up

Variable	Baseline	Follow-up	∆ mean	Cohen’s d
BPRS (score)	32.00 (21.00; 43.00)	18.00 (18.00; 23.50)	-10.92	-0.99***
SOD (pg/mL)	5416.39 ± 268.60	6226.69 ± 582.52	820.19	0.26**
S100B (pg/mL)	62.95 (27.33; 96.68)	64.14 (38.96; 103.83)	17.96	0.23*
Tryptophan (ng/mL)	2555.09 ± 178.24	1606.97 ± 166.84	-944.97	-0.81***
5-HT (ng/mL)	36.69 (20.05; 58.89)	15.19 (6.92; 31.336)	-18.53	0.69***
5-HIAA (ng/mL)	7.80 ± 0.48	3.09 ± 0.20	-4.61	-1.62***
KYN (ng/mL)	79.65 (66.80; 107.95)	54.52 (35.20; 78.46)	-22.81	-0.58***
KYNA (ng/mL)	1.21 (1.02; 1.65)	0.68 (0.47; 0.92)	-0.66	-0.72***
Cortisol (ng/mL)	2.47 (1.79; 3.06)	1.95 (1.20; 2.87)	-0.50	-0.34***
GSH (ng/mL)	0.11 (0.06; 0.17)	0.04 (0.02; 0.13)	-0.047	-0.28**
GSSG (ng/mL)	14.78 (7.09; 27.78)	24.82 (11.82; 55.56)	29.08	0.41***

Data at baseline and follow-up represent the mean ± 95% confidence interval (CI) when data were normally distributed, or median (25th and 75th quartile) when data were not normally distributed. ∆ = follow up - baseline, BPRS = Brief Psychiatric Rating Scale, SOD = superoxide dismutase; S100B = serum S100 calcium-binding protein B; 5-HT = serotonin, 5-HIAA = 5-hydroxyindoleacetic acid; KYN = kynurenine; KYNA = kynurenic acid; GSH = glutathione; GSSH = glutathione disulphide

Comparisons of baseline values and changes in BPRS scores and biomarker levels between recruitment sites (Site 1 vs. Site 2) indicated baseline differences, with participants from Site 1 showing higher baseline BPRS scores, tryptophan, 5-HT, 5-HIAA, KYN, KYNA, and GSH, whereas no significant differences were observed for SOD, S100B, cortisol, or GSSG (Supplementary Table 1). Over the follow-up period, differences in changes in BPRS scores and biomarker levels were observed between sites, with larger reductions in BPRS scores, tryptophan, 5-HT, 5-HIAA, and KYNA in Site 1 compared with Site 2 (all *p* < 0.001). Site 1 also showed decreases in SOD, KYN, and cortisol levels, while increases in these biomarkers were observed in Site 2. No significant between-site differences were found for S100B, GSH, or GSSG. However, the small and unequal sample sizes across sites limit the robustness of these between-site comparisons.

Figure 1 depicts cases where a weak, moderate, or strong correlation was found between the BPRS score at baseline and the indicated variable on the Y axis. In all other instances no significant correlation with the BPRS score at baseline was found (data not shown). As can be seen in Fig. 1A, there is a strong negative correlation between BPRS score at baseline and ∆BPRS (*r* = 0.689; *p* < 0.001). Figure 1B, C and D show a weak positive correlation between BPRS at baseline and the serum concentration of 5-HIAA (*r* = 0.217; *p* < 0.0198) KYNA (*r* = 0.293; *p* = 0.004) and GSSG (*r* = 0.231; *p* = 0.023).

It was important to investigate whether patient psychiatric symptomatology (i.e., ∆BPRS over three weeks) was associated with specific trends in corresponding baseline serum concentrations of the biomarkers and is depicted in Fig. 2. In all other cases no significant correlation with the ∆BPRS was found (data not shown). Weak to moderate negative correlations were evident between ∆BPRS and 5-HIAA baseline concentration (*r* = -0.410; *p* = 0.002), KYN baseline concentration (*r* = -0.326; *p* < 0.006) and baseline KYNA (*r* = -0.384; *p* = 0.006). In addition, partial correlations were performed while adjusting for sex, ethnicity, age, socio-economic status, and chronic medication use. However, these adjustments showed that no demographic factor affected the results obtained.

It was also important to investigate whether patient psychiatric symptom changes (as defined by the change in BPRS scores over the 3-week treatment period, i.e., ∆BPRS) was associated with any corresponding changes in biomarker concentrations over the same 3-week treatment period, i.e., ∆ biomarker serum level. Pearson correlations were performed to show the correlations between ∆BPRS and specific ∆biomarker serum levels, as depicted in Fig. 3. In all other cases no significant correlation with the ∆BPRS was found (data not shown). Weak to moderate positive correlations were evident between ∆BPRS and ∆tryptophan (*r* = 0.523; *p* < 0.001), ∆5-HT (*r* = 0.309; *p* = 0.007), ∆5-HIAA (*r* = 0.525; *p* < 0.001), ∆KYN (*r* = 0.567; *p* < 0.001), ∆KYNA (*r* = 0.485; *p* < 0.001), ∆cortisol (*r* = 0.372; *p* = 0.001) and ∆GSSG (*r* = 0.241; *p* = 0.036). Again, we adjusted for sex, ethnicity, age, socio-economic status, and chronic medication use and found that these adjustments did not influence the outcome of the results.

When stratified by study site (data not shown), several correlations between baseline BPRS scores and biomarkers, as well as between changes in BPRS scores and biomarkers, were observed within individual sites. In Site 2, a positive correlation was observed between baseline BPRS scores and baseline GSSG levels (*r* = 0.46, *p* = 0.001), while in Site 1 a weak negative correlation was observed between baseline BPRS scores and baseline SOD (*r* = -0.28, *p* = 0.049). A negative correlation between baseline BPRS scores and change in ΔBPRS was observed in Site 1 (*r* = -0.63, *p* < 0.001), whereas this association was not statistically significant in Site 2 (*p* = 0.12). Additional within-site associations included negative correlations between ΔBPRS and baseline 5-HT in Site 2 (*r* = -0.41, *p* = 0.02) and between ΔBPRS and ΔS100B in Site 1 (*r* = -0.49, *p* = 0.004), as well as a weak positive correlation between ΔBPRS and Δcortisol in Site 1 (*r* = 0.38, *p* = 0.03). No other statistically significant correlations were identified within either site. These within-site analyses should be interpreted cautiously given the small sample sizes and limited statistical power.

## Discussion

This study assessed the effect of SUD treatment on psychiatric symptoms (BPRS scores) and serum biomarkers (S100B, SOD, GSH, GSSG, kynurenine pathway metabolites, and cortisol) in a cohort of South Africans with SUD. Most participants were young Black African men from low socio-economic backgrounds, reflecting the ethnic distribution of the South African population at large [25], and recent findings from treatment centres in the North-West province [6, 7]. Majority of participants engaged in polysubstance use, with only a small proportion reporting single-substance use, a pattern aligning with findings from the North West province, where polysubstance abuse accounts for approximately 69% of all substance use disorder cases in the region [7].

The main findings were: (a) most participants’ BPRS scores decreased from day 0 to day 21, with larger effects at higher baseline scores; (b) serum concentrations of most biomarkers decreased over time, except for SOD, S100B, and GSSG, which increased; and (c) correlations were found between 3-week BPRS changes and baseline levels of 5-HIAA, KYNA, GSSG, as well as changes in select biomarkers, including tryptophan, 5-HT, 5-HIAA, KYN, KYNA, GSSG, and cortisol.

Although site-stratified analyses suggested differences in baseline characteristics, symptom change, and biomarker trajectories between sites, these findings should be interpreted with caution given the small and uneven sample sizes, which limit statistical power and raise the possibility that observed differences may reflect baseline imbalances rather than true site-specific effects, although correlations involving change scores inherently incorporate baseline values.

### Participants exhibited severe psychiatric symptoms upon admission and showed improvement after three weeks of substance use disorder (SUD) treatment

A decrease in BPRS scores over the follow-up period suggests that the 3-week treatment improved psychiatric symptoms, with participants who had higher baseline scores showing greater improvement. This may indicate a positive psychological response to SUD treatment. It is currently not known whether the response of SUD patients with a lower BPRS score may be associated with altered levels of select/specific biomarkers only, as discussed in the paragraphs below. Interviews with the attending psychiatrists and staff at the three facilities suggested that standard treatment included medication (i.e., paracetamol, thiamine, cyclizine, haloperidol, phenytoin, clozapine, and/or sodium valproate) and psychotherapy (e.g., group and individual sessions). In this regard studies have shown that treatment with medication for withdrawal symptoms, relapse, and psychiatric comorbidity together with cognitive behavioural therapy can lead to an improvement in clinical outcome of the patient [26–28]. SUD is often associated with mental health co-morbidities, which commonly include psychotic symptoms [29]. Furthermore, Madero et al., (2020) [30] reported that patients with schizophrenia or psychotic disorders that cannabis users, had more severe psychopathology (i.e., higher BPRS scores) in the week leading up to admission compared to people with depression, bipolar disorder, or addiction disorders. This underscores the importance of thoroughly assessing the severity of psychiatric symptoms in patients admitted for SUD to ensure that observed improvements (or lack thereof) reflect a true therapeutic response rather than solely a regression from initially higher baseline severity or the persistence of undiagnosed or untreated comorbid conditions.

### Selected biomarker serum levels increased or decreased from baseline to follow-up

#### Biomarkers of oxidative stress increased from baseline to follow-up

The current study found an increase in oxidative stress biomarkers (SOD and GSSG) and a decrease in GSH from baseline to the 3-week follow-up. This may suggest that the SUD patients may have developed a mild increase in oxidative stress during the 3-week treatment period. One would typically expect these levels to decline with clinical improvement. However, the observed elevations may indicate that some participants were still experiencing physiological effects consistent with the withdrawal phase at the 3-week follow-up. This interpretation is supported by similar findings observed in patients with alcohol use disorder (AUD), where SOD levels were elevated during withdrawal [31]. Previous studies show that after cocaine withdrawal, GSH and SOD levels increase, indicating reduced oxidative stress [32, 33]. Since many participants used multiple substances, each may have contributed to elevated oxidative stress markers. Some were also treated with haloperidol, which has been linked to oxidative stress and GSH reduction over time [34, 35]. In contrast, one preclinical study found that haloperidol and clozapine had no effect on SOD concentrations [36]. Further research is needed to confirm the clinical significance of these biomarkers in SUD treatment, as lower GSH levels can increase oxidative stress [37] while severe stress may deplete GSH by preventing the reduction of GSSG to GSH [38]. Our findings therefore suggests that oxidative stress may have been present throughout the entire 3-week SUD treatment period.

#### Serum calcium-binding protein B (S100B) increased from baseline to follow-up

S100B levels increased from baseline to follow-up, though with a small effect size. In children, S100B acts as a neurotrophic factor, but in adults, elevated levels are linked to traumatic brain injury, neurodegeneration, lesions, or infection [39]. At high levels, S100B may be neurotoxic, but it has protective effects at lower concentrations [40, 41]. The current findings may indicate astroglial activation or other brain-related changes that occur in addiction, as previous studies found that chronic alcohol use can lead to glial cell activation in the brain and chronic alcohol use can lead to an increase in S100B [42–44]. This contrasts with findings of another study in AUD, where S100B levels decreased over time [45], which supports the idea that withdrawal may still be at play (see limited cortisol response below) and/or may indicate that S100B levels fluctuates during the treatment period. These aspects warrant further investigation, especially in polysubstance users.

#### Biomarker of stress decreased from baseline to follow-up

Participants’ cortisol levels decreased significantly over the 3-week treatment period. Participants are usually anxious at the point of admission due to stress factors like uncertainty and apprehensive thoughts associated with no longer being able to use the substance. The reduction in cortisol suggests that the treatment interventions at the respective facilities were successful in reducing stress, though the small effect size suggest that recovery may not have reached the desired degree of remission yet (due to the short treatment duration). In individuals with depression, often correlated with SUD, a dysfunctional hypothalamic-pituitary-adrenal (HPA) axis and elevated cortisol levels are common. Stress and high glucocorticoid levels contribute to brain reward dysfunction, escalating drug use as the individual moves from occasional drug use to drug dependency. High amounts of glucocorticoids and stress peptides associated with addiction generate an internal form of stress, characterized by anxiety-like behaviours during the addicted state [13], which may explain elevated levels of cortisol at baseline. Exposure to certain stressors or reminders of these stressors can also trigger substance use and increase anxiety. Another study found lower cortisol levels in SUD patients after 40 days of treatment suggesting quicker HPA axis recovery following withdrawal compared to other neurobiological systems [46].

#### Kynurenine pathway and its metabolites are affected by SUD treatment

Tryptophan, 5-HT, 5-HIAA, KYN, and KYNA significantly decreased from baseline to follow-up, with 5-HIAA showing the largest practical significance, suggesting it could be a robust biomarker for SUD-related psychiatric symptom improvement. Although early work primarily focused on opioids and cocaine, emerging evidence indicates that a broader range of substances including alcohol, methamphetamine, cannabis, nicotine and ketamine, may influence kynurenine pathway metabolism through inflammatory and neuroimmune mechanisms [47]. These substances have generally been associated with a shift toward the neurotoxic branch of the pathway (e.g., increased quinolinic acid and reduced KYNA), although findings are not entirely consistent. However, compared to other neurobiological systems, the literature remains limited and heterogenous across substances, populations and study designs. Preclinical research indicates that acute administration of amphetamine decreases KYNA levels in the rat striatum. This effect is prevented by haloperidol, an antipsychotic dopamine_2_ receptor antagonist [48], indicating a potential role for dopamine in inflammatory processes during withdrawal. Similarly, a rodent model for ethanol dependence and withdrawal revealed that chronic ethanol exposure may induce neuroimmune activation, triggering KYN pathway activation [49].

Studies conducted on cocaine use disorder and AUD found observed decrease in the neuroprotective metabolite KYNA after three weeks of detoxification, consistent with our findings [50, 51]. Decreased KYNA has also been linked to substance abuse [52]. Given the significance of serotonin transporter (SERT) in controlling 5-HT concentration (understanding that 5-HT and the kynurenine pathway are linked via its common precursor tryptophan) in specific compartments and the strong association between platelet SERT and plasma 5-HT, it is fair to assume that platelet SERTs are involved in elevated plasma 5-HT levels in abstinent patients [50]. Unlike other studies that observed only KYNA alterations, our study found changes in tryptophan, KYN, 5-HT, and 5-HIAA. Changes in quinolinic acid were below detection limits, preventing further investigation. An imbalance in the kynurenine pathway can lead to decreased 5-HIAA, a metabolite of 5-HT, and this decrease has also been linked to major depressive disorder [53, 54]. Our study also showed declines in both 5-HT and 5-HIAA, suggesting potential neurochemical alterations associated with psychiatric comorbidities [55–57], as also indicated by the improvement observed in psychiatric symptomatology in our cohort.

### Psychiatric distress at baseline correlated with biomarkers at baseline

Higher BPRS scores at baseline (i.e., significant psychiatric symptomatology, either due to intoxication / withdrawal / due to substance induced disorders or due to comorbid SUD and psychiatric illness) were associated with a greater reduction in BPRS scores (negative ∆BPRS, indicative of improved SUD psychiatric symptomatology), suggesting that severely distressed SUD patients may respond better to treatment than those with less distress. Some patients with lower baseline BPRS scores still responded to treatment, and it is hypothesized that changes in certain biomarker concentrations could reflect this response. This study uniquely investigates the relationship between baseline psychiatric symptomatology and subsequent changes in SUD over three weeks. Additionally, we found no other studies examining the correlation between baseline biomarker levels and BPRS scores in SUD patients. Our findings suggest that high baseline BPRS scores at admission are positively correlated with high serum levels of 5-HIAA, KYNA, and GSSG. While KYNA is linked to glutamatergic modulation and cognitive processes, the relevance of 5-HIAA and GSSG to glutamatergic function or neurological stress is less well-established [20, 58, 59]. These findings should therefore be interpreted as preliminary evidence of associations rather than evidence of specific neurobiological mechanisms, though they are consistent with cognitive challenges reflected in higher baseline BPRS scores.

Notably, ∆BPRS scores negatively correlated with 5-HIAA, KYN, and KYNA levels at baseline, suggesting that higher levels of these biomarkers may predict a more significant improvement in psychiatric symptoms, potentially leading to a greater decrease in BPRS scores after three weeks. Overall, our findings imply that higher initial BPRS scores and biomarker levels may be associated with a more substantial change in psychiatric symptoms following treatment, highlighting the potential predictive value of these biomarkers for psychiatric symptom improvement in SUD patients.

### Change over time in psychiatric symptoms correlated with change over time in biomarker levels

The improved psychiatric symptoms following treatment (indicated by negative ∆BPRS scores) were associated with reduced serum cortisol levels, suggesting a decrease in patients’ biological stress and anxiety during SUD treatment. However, positive correlations between ∆BPRS score with ∆tryptophan, ∆5-HT, ∆5-HIAA, ∆KYN and ∆KYNA in this study may indicate that biomarkers associated with worsening psychiatric symptomatology may still be under influence of the unresolved withdrawal phase, since the levels of these markers have previously been linked to other disorders such as depression and anxiety, of even a reduced BPRS score with bipolar mania [54]. Therefore, our findings may suggest that patients still presented with some degree of neurobiological distress and stress (i.e., cortisol) after 3 weeks of treatment. Alterations in these biomarkers, along with ∆BPRS, may suggest that longer treatment or adjustments may be needed for effective management. Additionally, patients with minimal changes in BPRS scores may be at risk for future psychiatric illnesses, highlighting the importance of early preventative strategies.

## Conclusion

Limitations of this study include the omission of other biomarkers, such as inflammatory cytokines, and the limited diversity of participants due to the study being conducted at three specific facilities in the North West province, South Africa. The patient profiles admitted for SUD treatment restricted variation in ethnicity, sex, and substance type, thereby limiting the generalizability of the findings to the broader South African or global population, but rendering it a useful pilot and reflective of patients typically treated at these facilities and from the geographical area they serve. Most participants were polysubstance users, with relatively few reporting single-substance use. Substance use histories were obtained through self-report during clinical interviews; however, given the voluntary nature of disclosure, it is possible that some participants did not report all substances used, for example due to concerns about stigma or social consequences. In addition, individual substance profiles were not consistently or clearly documented in the available clinical records, limiting the level of detail accessible to the research team. Consequently, participants could not be reliably stratified by specific substance type, which constrained the analyses and the strength of inferences that could be drawn. As the biomarkers investigated may be independently influenced by both substance type and abstinence status, this combination of incomplete disclosure and limited documentation likely contributed to variability in biomarker outcomes and should be considered when interpreting the findings. The limited and heterogeneous literature across substances represents an additional limitation for substance-specific interpretation. Future analyses could benefit from grouping participants according to treatment regimens. The current study looked at treatment over a 3-week period, whereas important biomarker changes may only be seen at a later stage, following 6-weeks (or longer) of withdrawal. It is therefore recommended that future studies consider extending the timeline of the study. Information on the specific medication prescribed was available for only 33 participants; therefore, differences in treatment could not be fully accounted for in the statistical analysis. Nevertheless, since each participant served as their own control and treatment protocols were generally standardized within facilities, overall trends in psychiatric symptom improvement could still be observed. Although some variation in treatment practices between facilities may have occurred, similar staged detoxification approaches were used at each facility and is unlikely to have meaningfully influenced the primary outcomes. However, participant distribution across sites was highly uneven, which limited the feasibility of conducting adequately powered site-stratified analyses. One site contributed only two participants (Site 3) and was therefore not suitable for meaningful comparative analyses, while the remaining sites (Sites 1 and 2) had small sample sizes. These constraints resulted in insufficient statistical power to conduct robust within-site correlation analyses or to reliably detect between-site differences. In addition, observed differences in changes in biomarkers between the two larger sites should be interpreted with caution, as they may be partly attributable to baseline differences, with one site demonstrating higher baseline levels and consequently larger absolute reductions, rather than reflecting true differences in treatment response between sites. Overall, site-level findings should be considered exploratory. The clinical implication of the BPRS is not always easy to interpret, therefore the addition of a specific measure of SUD treatment response of specific psychometric parameters thereof together with the BPRS can support the interpretation of the BPRS results in relation to the SUD treatment success. The BPRS evaluates five symptom domains on a 7-point scale, but the current study did not explore correlations between these symptom-level domains and biomarkers, suggesting the need for more targeted psychiatric assessments focusing on specific symptoms.

This study introduced a novel approach by measuring serum levels of tryptophan, 5-HT, 5-HIAA, KYN, KYNA, and GSSG alongside BPRS scores. Over a 21-day period, patients’ psychiatric symptoms improved according to BPRS scores. Notably, baseline serum levels of 5-HIAA, KYN, and KYNA correlated with changes in BPRS scores, indicating the kynurenine pathway’s potential clinical value in predicting psychiatric symptom changes during SUD treatment. However, further research is needed on these biomarkers and their serum/plasma level changes. The growing awareness of the need for better SUD treatments highlights the importance of correlating psychiatric symptoms with biomarker serum levels to enhance understanding of SUD’s neurobiological basis and inform optimal treatment strategies. While serum levels of many biomarkers decreased over the 3-week period, some, including SOD, S100B, and GSSG, increased, suggesting that some patients may still be in the withdrawal phase. This indicates that treatment duration may need to be extended for certain individuals. Therefore, future studies should consider measuring blood levels at a later stage to assess biomarker changes and verify treatment response.

## Supplementary Information

Below is the link to the electronic supplementary material.

Supplementary Material 1

## Data Availability

All data generated or analysed during this study are included in this published article.

## Abbreviations

5-HT: Serotonin
5-HIAA: 5-hydroxyindoleacetic acid
AUD: Alcohol use disorder
BPRS: Brief Psychiatric Rating Scale
CI: Confidence interval
DSM-5: Diagnostic and Statistical Manual of Mental Disorders
ELISA: Enzyme-linked immunosorbent assay
GSH: Glutathione
GSSG: Glutathione disulphide
HPA: Hypothalamic-pituitary-adrenal
KYN: Kynurenine
KYNA: Kynurenic acid
LCMS: Liquid chromatography mass spectrometry
NWU-HREC: North-West University Health Research Ethics Committee
NWDoHRC: North West Department of Health Research Committee
SACENDU: South African Community Epidemiology Network on Drug Use
SERT: Serotonin transporter
SOD: Superoxide dismutase
SPSS: Statistical Package for the Social Sciences
SUD: Substance use disorder
UNODC: United Nations Office on Drugs and Crime
VTA: Ventral tegmental area
WHO: World Health Organisation
