# Supplementary material for: Neuroinflammatory-related biomarkers and psychiatric symptom changes in substance use disorder: a South African cohort study
Source: BMC Psychiatry. 2026 May 30;26:598. doi: 10.1186/s12888-026-08237-0 (PMC13445686; doi:10.1186/s12888-026-08237-0)
Supplement: Supplementary file 1 [file 12888_2026_8237_MOESM1_ESM.docx]

**Supplementary Table 1:** **BPRS scores and serum biomarker concentrations in the study population at baseline, as well as changes over time, stratified by study site (Site 1 vs Site 2).** Data represent the mean ± 95% confidence interval (CI) when data were normally distributed, or median (interquartile range) when data were not normally distributed. ∆ = follow up - baseline, *BPRS = Brief Psychiatric Rating Scale, SOD = superoxide dismutase; S100B = serum S100 calcium-binding protein B; 5-HT = serotonin, 5-HIAA = 5-hydroxyindoleacetic acid; KYN = kynurenine; KYNA = kynurenic acid; GSH = glutathione; GSSH = glutathione disulphide.*

| **Variable** | **Site 1 at baseline (mean)**  **N = 53** | **Site 2 at baseline (mean)**  **N = 47** | **p-value** | **Cohen’s d** |
| --- | --- | --- | --- | --- |
| BPRS (score) | 38.80 (35.64; 41.97) | 27.34 (23.05; 31.63) | <0.001 | 0.88 |
| SOD (pg/mL) | 5136.24 ± 2075.33 | 5298.91 ± 2004.75 | 0.465 | -0.14 |
| S100B (pg/mL) | 76.50 (60.73; 92.26) | 58.27 (44.18; 72.35) | 0.086 | 0.34 |
| Tryptophan (ng/mL) | 2673.91 ± 1331.04 | 2237.55 ± 1087.63 | 0.004 | 0.53 |
| 5-HT (ng/mL) | 46.95 (39.63; 54.26) | 34.89 (24.46; 41.32) | 0.015 | 0.49 |
| 5-HIAA (ng/mL) | 9.27 ± 2.59 | 6.71 ± 3.16 | <0.001 | 1.30 |
| KYN (ng/mL) | 91.29 (83.62; 98.95) | 76.06 (68.30; 83.81) | 0.006 | 0.56 |
| KYNA (ng/mL) | 1.82 (1.56; 2.07) | 1.00 (0.90; 1.09) | <0.001 | 1.14 |
| Cortisol (ng/mL) | 2.67 (2.34; 3.00) | 2.59 (2.27; 2.92) | 0.740 | 0.07 |
| GSH (ng/mL) | 0.15 (0.13; 0.18) | 0.10 (0.08; 0.13) | 0.005 | 0.57 |
| GSSG (ng/mL) | 26.79 (19.30; 34.27) | 17.55 (11.11; 24.00) | 0.064 | 0.37 |
|  | **Site 1 ∆ (mean)**  **N = 42** | **Site 2 ∆ (mean)**  **N = 36** | **p-value for ∆** | **Cohen’s d** |
| **∆**BPRS (score) | -17.03 (-20.46; -13.59) | -4.16 (-5.81; -2.51) | <0.001 | -1.52 |
| **∆**SOD (pg/mL) | -956.33 ± 5320.45 | 1959.98 ± 2760.98 | <0.001 | -0.85 |
| **∆**S100B (pg/mL) | 6.27 (-22.23; 34.76) | 26.53 (5.49; 47.57) | 0.257 | -0.25 |
| **∆**Tryptophan (ng/mL) | -1696.45 ± 1128.41 | -150.01 ± 30.44 | <0.001 | -1.72 |
| **∆**5-HT (ng/mL) | -31.38 (-38.30; -24.47) | -5.53 (-13.94; 2.88) | <0.001 | 1.09 |
| **∆**5-HIAA (ng/mL) | -6.51 ± 2.85 | -2.97 ± 3.46 | <0.001 | -1.64 |
| **∆**KYN (ng/mL) | -47.87 (-57.91; -37.83) | 2.79 (-6.68; 12.25) | <0.001 | -1.67 |
| **∆**KYNA (ng/mL) | -1.22 (-1.50; -0.93) | -0.10 (-0.25; 0.57) | <0.001 | -1.53 |
| **∆**Cortisol (ng/mL) | -1.29 (-1.61; -0.96) | 0.20 (-0.26; 0.66) | <0.001 | -1.22 |
| **∆**GSH (ng/mL) | -0.06 (-0.11; -0.02) | -0.01 (-0.05; 0.29) | 0.087 | -0.84 |
| **∆**GSSG (ng/mL) | 16.23 (0.73; 31.73) | 44.48 (15.64; 73.33) | 0.086 | -0.40 |
